# Patient-identified priorities for a mental health program tailored to premenstrual dysphoric disorder

**DOI:** 10.1177/17455057251331697

**Published:** 2025-06-13

**Authors:** Sneha Chenji, Christine Bueno, May Ly, Sandi MacDonald, Rebekka Chartier, Catherine Lunn, Jennifer L. Gordon

**Affiliations:** 1 Department of Psychology, University of Regina, Regina, SK, Canada; 2 International Association for Premenstrual Disorders (IAPMD), Boston, MA, USA; 3 Patient Advisory Panel, Regina, SK, Canada

**Keywords:** premenstrual dysphoric disorder, premenstrual disorders, patient-centered, psychotherapy, self-help intervention

## Abstract

**Background:**

There are no mental health programs tailored to premenstrual dysphoric disorder (PMDD).

**Objectives:**

Examine the content and features that PMDD patients desire in a mental health program.

**Design:**

Anonymous online survey.

**Methods:**

A survey, co-developed with PMDD patients, was distributed to followers of the International Association for Premenstrual Disorders (IAPMD) on social media. Respondents rated the importance of addressing various PMDD symptoms, psychosocial consequences, psychoeducational topics, and their preference for various techniques and features.

**Results:**

In total, 308 respondents rated suicidality, feelings of hopelessness and anxiety, and impacts on one’s relationship with one’s partner and children as important content areas for a PMDD-tailored mental health program. Providing evidence-based information and teaching techniques for managing distressing thoughts and emotions were also highly desired.

**Conclusion:**

Our findings help inform the development of a new mental health program that specifically addresses the unique emotional and psychosocial challenges associated with PMDD.

## Plain Language Summary

What do PMDD patients need in a mental health program?

Premenstrual dysphoric disorder (PMDD) affects 1-3% of menstruating individuals and is characterized by cyclical psychiatric symptoms for 1-2 weeks before the start of a period. There are no mental health programs tailored to PMDD. Through an anonymous online survey, co-developed with patients, we examined the content and features that PMDD patients desire in a mental health program. Analysis of 308 survey respondents show that suicidality, feelings of hopelessness and anxiety, and impact on one’s relationship with one’s partner and children were rated as important content areas for a PMDD-tailored mental health program. Survey results also show that evidence-based information and techniques for managing distressing thoughts and emotions were highly desired. These findings highlight patient-informed needs that can help in the development of a new mental health program, specifically addressing the unique challenges associated with PMDD.

## Introduction

Premenstrual dysphoric disorder (PMDD), affecting 1%–3% of menstruating individuals, is characterized by the cyclical presence of psychiatric symptoms in the luteal phase of the menstrual cycle that resolves in the week following menstruation.^1^ Though increased affective sensitivity to ovarian hormone fluctuation across the menstrual cycle has been shown to play a key role in the etiology of PMDD,^2^ there is increasing recognition that psychotherapy is also a reasonable treatment approach to target the affective symptoms of PMDD.^3^ To date, cognitive behavioral therapy (CBT) has received the most attention as a treatment for PMDD, with two randomized trials finding it to be effective in improving symptoms.^4,5^

The International Association for Premenstrual Disorders (IAPMD) is a patient-led non-profit organization aimed at empowering those living with premenstrual disorders through peer support, education, research, and advocacy. A current project of IAPMD involves developing a PMDD-specific self-guided psychotherapy intervention that addresses the unique challenges of living with PMDD. After all, research suggests that effectively tailoring psychotherapeutic approaches to specific disorders results in increased treatment efficacy.^6^ With this goal in mind, IAPMD developed and launched an online survey on its social media platforms asking respondents what they would hope to see included in such a program, specifically: (1) the relative importance of targeting the various PMDD symptoms and interpersonal challenges associated with PMDD; (2) preference for specific techniques to improving symptoms; (3) the relative importance of psychoeducational topics relevant to PMDD; and (4) preference for program format. We report the results of this survey here.

## Methods

In addition to three team members affiliated with IAPMD (SM, SC, and JLG), two patients with lived experience (CL and RC) were recruited to co-develop the survey to ensure that survey questions were clear and lists of possible items were comprehensive. The final anonymous survey (see Supplemental Material) was estimated to take approximately 15–20 minutes to complete. It was administered online via Qualtrics (Provo, UT) and included the following blocks: (A) Summary of the purpose/content of the survey and assessment of respondent eligibility. Participants were eligible to complete the survey if they had been diagnosed with or indicated the presence of a premenstrual disorder. Those who indicated that they had not were skipped to the end of the survey. (B) Demographic information: country of residence, age, gender identity, race, highest level of education completed, household income, and history of participating in psychotherapy; (C) Respondent preferences related to the following: psychoeducational topics related to premenstrual disorders, symptoms to target in a PMDD-focused intervention, PMDD-related psychosocial consequences to address in such an intervention, and techniques to be used; (D) Preference for intervention features (e.g., self-help exercises, reminders, completion of the program with peers); and (E) Preference for program format (e.g., content type, frequency). A 5-point Likert scale was used to assess responses for perceived importance and preference, from 1 = “not at all important” to 5 = “extremely important.” Non-response items were provided where possible.

Our survey flow included adaptive questioning allowing us to ensure that collection of information was tailored to participants’ responses (e.g., asking about types of therapy participants were exposed to if participants indicate a history of psychotherapy). To ensure ease of response and completion, number of items was limited to 1–3 items per page, and the overall survey was distributed across 17 pages. Participants had the option to change their responses through a ‘Back’ button.

This survey was based on convenience sampling and was accessible to anyone with the survey link. The option to prevent multiple responses was selected so that participants could submit the survey only once. The survey was distributed by IAPMD through its social media channels—Facebook, Instagram, LinkedIn, and X (formerly Twitter)—and was available to participants for a duration of 3 weeks (March 4th to 21st, 2024). Though there was no set goal regarding sample size, it was hoped that at least a few hundred responses would be achieved to ensure the responses were generalizable to the overall population of IAPMD followers. Participants were not offered any incentives for completing the survey. The reporting of this study conforms to the CHERRIES guidelines.^7^

## Statistical analysis

We computed descriptive statistics such as mean age and reported age for onset of symptoms, median education and income, percentages for country of residence, ethnicity, self-identified gender, and current treatment use. We calculated the percentage of participants indicating high preference or considered extremely important to address (Likert score > 4), as well as the mean and standard deviation of the Likert scores for each of the survey items.

## Results

The survey was completed by 354 participants; however, respondents were included in the analysis only if one of the following conditions was met: (1) they had been diagnosed with premenstrual disorder by a medical professional; (2) they had confirmed their PMDD via prospective daily ratings for at least two menstrual cycles; or (3) they reported prior severe premenstrual symptoms and experienced significant relief upon taking continuous birth control or a GnRH analog treatment. This resulted in 308 respondents being included in the current analysis. Incomplete surveys, that is, where responders skipped a question were considered for analysis. The mean response time for survey completion was *M(SD)* = 21.0 (75.0) minutes.

### Participant characteristics

The majority of respondents resided in one of five countries: 33% in the United States, 19% in Canada, 19% in the United Kingdom, 7% in Australia, and 5% in the Netherlands. Participants mean age was *M*(SD) = 36.7 (7.8)  years and reported the onset of their PMDD symptoms to be at the mean age of *M*(SD) = 23.0 (9.2)  years. About 93% of respondents identified as a woman, while the remainder identified as non-binary/two-spirited (and missing information in 3%). Most (84%) respondents identified as White, 5% indicated multiple racial backgrounds, 4% as Asian, 3% as Latino, 1% as Indigenous, and 2% other. The median education level was a completed a bachelor’s degree; 58% indicated that they had a university degree. Median household income was between $70,000 and $79,999. In considering the history of therapy, 55% reported having received psychotherapy in the past, and 33% were currently in therapy.

Regarding current use of PMDD treatments, 47% indicated that their PMDD symptoms were untreated; 38% reported experiencing partial relief with treatment; 5% reported complete relief with the use of medications, including selective serotonin reuptake inhibitors, continuous birth control, or hormone replacement therapy; and 7% reported that they had undergone natural/chemical/surgical menopause and therefore no longer experienced PMDD symptoms.

### Psychotherapy needs

**Figure 1.**
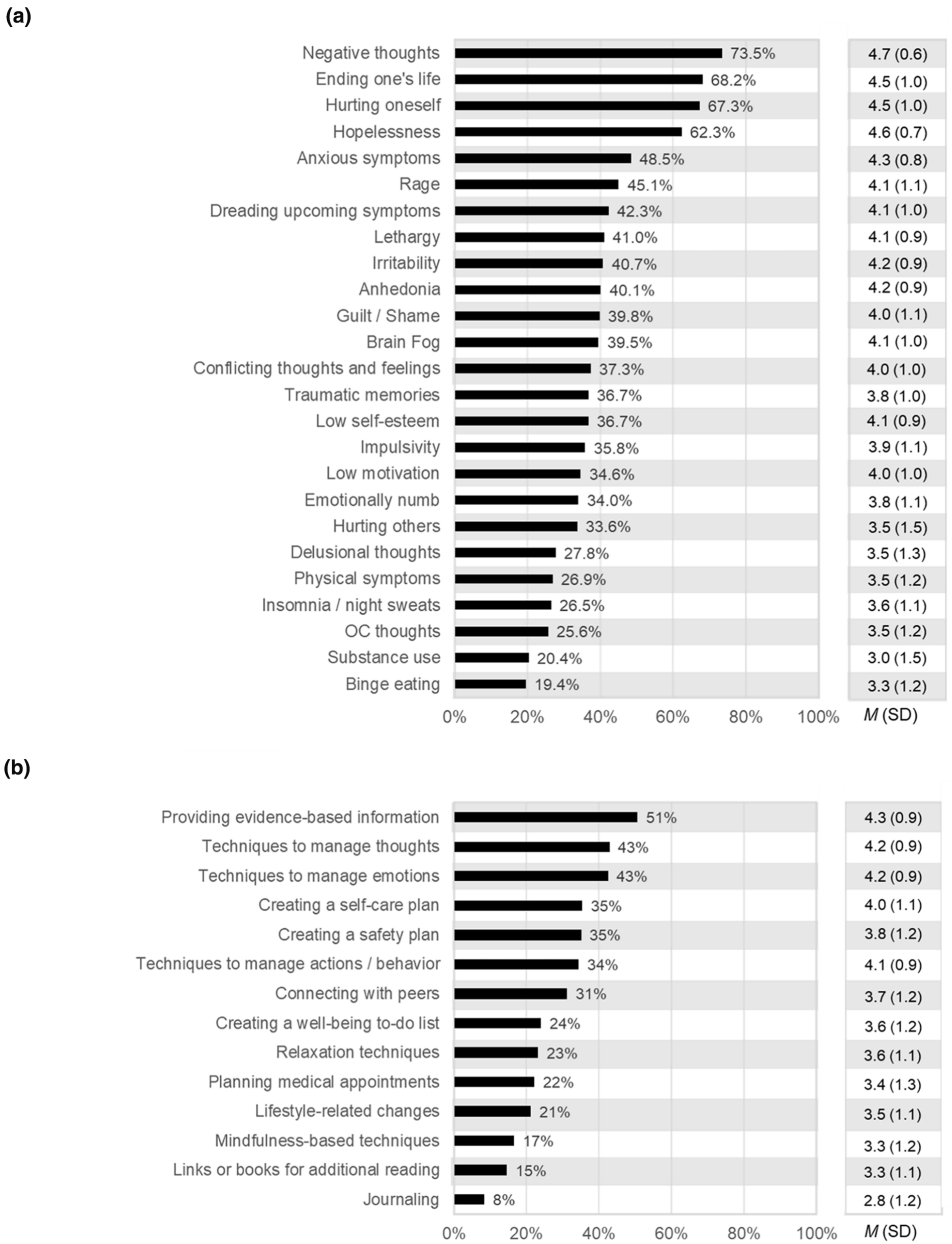
Percentage of respondents selecting “Extremely Important” (Likert score = 5) for addressing: (a) symptoms associated with PMDD, and (b) techniques preferred in a PMDD program.

Of the symptoms to be addressed in a PMDD-specific intervention, the top five symptoms considered “extremely important” to address by most participants (% rating > 4 and mean ratings) were: extreme negative thoughts (73%) *M*(SD) = 4.7(0.6), thoughts of hurting oneself (69%) *M*(SD) = 4.5(1.0), thoughts of ending one’s life (68%) *M*(SD)  = 4.5(1.0), hopelessness (63%) *M*(SD)  = 4.6(0.7), and anxious symptoms (48%) *M*(SD)  = 4.3(0.8) (Figure 1A). Of the types of skills or techniques to be included in such a program, the top five that were highly preferred by most participants (% ratings > 4) included: being provided with evidence-based information (51%) *M*(SD)  = 4.3(0.9), techniques to manage emotions (43%) *M*(SD)  = 4.2(0.9), techniques to manage thoughts (43%) *M*(SD)  = 4.2(0.9), creating a self-care and safety plan (35%) *M*(SD)  = 3.9(1.1), and techniques to manage actions /behavior (34%) *M*(SD) = 4.1(0.9) (Figure 1B).

Of possible topics related to interpersonal relationships to be considered for a PMDD-tailored program, one’s relationship with a romantic partner, relationship with children, and interacting with healthcare providers were deemed “extremely important” (Likert score > 4, Table 1A). In terms of other content to consider for inclusion, awareness of treatment options that have strong scientific evidence, understanding the impacts of life stage on PMDD symptoms, understanding their rights in the workplace, learning about treatment options with promising evidence, and an overview of hormones and the menstrual cycle were all highly rated (Table 1B). Additionally, patients indicated a high preference for features such as access to a professional, integrated symptom tracking, sharing humor-related content, inclusion of self-help exercises, and access to individual peer support (Table 1C). Finally, for program format, participants endorsed a self-paced format including audio-visual content, with new content made on a weekly basis (Table 1D).

**Table 1.** Percentage of respondents selecting “Extremely Important,” mean and standard deviation (SD) of Likert scores for social aspects, information, feature, and format preferences in a PMDD-focused program.

Survey response	Rating > 4 ‘Extremely Important’	Likert score
	%	M (SD)
A. Social aspects to be addressed		
Relationship with romantic partner	59%	4.5 (0.9)
Relationship with children	53%	4.2 (1.2)
Interacting with healthcare providers	45%	4.1 (1.1)
Relationship with friends	32%	3.9 (1.0)
Relationship with family	31%	4.0 (1.0)
Interacting with colleagues	30%	3.9 (1.1)
Navigating social life	30%	3.9 (1.0)
B. Information to include		
Treatment options with strong evidence	66%	4.7 (0.6)
Impacts of life stage on symptoms (e.g., pregnancy, menopause transition)	49%	4.4 (0.8)
Rights in the workplace	47%	4.2 (1.1)
Treatment options with promising evidence	45%	4.3 (0.8)
Overview of hormones and the menstrual cycle	41%	4.3 (0.9)
Diagnostic criteria for PMDD and PME	39%	4.1 (1.0)
Molecular changes underlying symptoms	38%	4.1 (1.0)
Risk factors for PMDs	36%	4.0 (1.0)
Symptom tracking and scoring	36%	4.1 (0.9)
Lifestyle changes to help symptoms	34%	4.0 (1.0)
Genetic contributions to PMDs	33%	4.0 (1.0)
Treatment options with limited evidence (e.g., supplements)	17%	3.1 (1.2)
C. Feature preferences		
Access to a professional	51%	4.4 (0.9)
Integrating symptom tracking in the program	32%	4.0 (1.0)
Humor-related content (e.g., memes)	30%	3.7 (1.2)
Self-help exercises	26%	3.8 (1.1)
Individual peer support	25%	3.6 (1.2)
Infographics	24%	3.7 (1.1)
Group peer support	24%	3.5 (1.2)
Reminders	20%	3.4 (1.2)
Video testimonials	18%	3.3 (1.2)
Written testimonials	13%	3.3 (1.1)
D. Program format preferences		
D1. Content type		
Video	29%	3.7 (1.2)
Audio	22%	3.8 (0.9)
Written	14%	3.2 (1.3)
D2. Duration ^a^		
Self-paced	49%	–
8–12 weeks duration	15%	–
<8 weeks	13%	–
D3. Frequency^a^		
Once a week	48%	–
Self-guided	15%	–
Once a month	8%	–
Daily	5%	–
Twice a month	3%	–

PMD: premenstrual disorder.

a Preferences for program duration and frequency are displayed as percentage of responders choosing their most preferred format.

## Discussion

This study explored what content and features should be included in a PMDD-tailored self-help mental health intervention. Results suggest that addressing extreme negative thoughts, suicidal thoughts and behaviors, as well as affective symptoms, such as hopelessness and anxiety, are high priorities for those with PMDD. Our findings also indicate a preference for techniques to manage emotions, thoughts, and behaviors, and actionable strategies such as creating a safety plan. These patient-identified priorities align with the need for better psychological support in PMDs.^8^ Previous research found that CBT is an effective strategy for improving PMDD severity by increasing recognition of negative thought patterns which occur during the premenstrual phase and teaching skills to counter these thought patterns.^4,5^ Moreover, dialectical behavior therapy (DBT) techniques have been shown to alleviate distress from extreme negative emotions and reduce suicidal behavior in individuals with borderline personality disorder.^9^ These findings conceptually support the possibility of combining techniques from CBT and DBT for a PMDD-focused program.

Our results also indicate a high preference for evidence-based information and features such as access to a healthcare professional and integrating symptom tracking. These findings are similar to the outcomes of a recent survey on preferences and intention to use an app for premenstrual mental health symptoms.^10^ We further expand on user preferences by capturing the desire to address suicidal thoughts and behaviors, as well as examining the preferences of specific topics of psychoeducation. This is important because a recent population-based cohort study indicated that people with premenstrual disorders were two times more likely to die of suicide than their unaffected peers^11^ and was also identified as a top research priority by patients.^8^ We also noted a high preference for addressing the consequences of PMDD symptoms on relationships with romantic partners and children, highlighting the need for alleviating conflicts with loved ones.^10,12^ Finally, we found a strong desire in the PMDD community to understand the impacts of life stages on PMDD symptoms and recognize rights in the workplace.

The findings described here have been carefully considered to develop a self-help program which consists of 11 animated explainer videos paired with voiceover. In response to the high endorsement for techniques to manage negative emotions and urges for suicide and/or self-harm, the first half of the program includes six modules focused on distress tolerance using DBT techniques. A dedicated module for creating a safety plan with step-by-step guidance and examples was also included. Next, in considering the endorsement for techniques to manage negative thoughts, the second half of the program includes five modules focused on CBT techniques, particularly cognitive restructuring. The modules also include guidance on when to use specific skills, depending on the situation and the intensity of their symptoms. Considering the patient-identified priority to address relationships with romantic partners and children, many of the examples included in the modules emphasize those relationship dynamics. The script for the modules underwent multiple rounds of feedback and editing. Additionally, each module will include completing some homework to help individuals learn more about their unique symptom patterns, practice techniques for managing symptoms and make a strategic plan for upcoming premenstrual phases. An initial version of the program was developed for a pilot study testing its effectiveness for reducing self-reported PMDD symptoms (NCT06648382).

The decision to create a self-directed program rather than a clinician’s treatment manual for in-person treatment was based on several factors. First, a prior study had already found internet-based CBT to be effective for the treatment of PMDD.^5^ Second, we reasoned that this format could be most effectively scaled up and made accessible to those with limited access to qualified mental health professionals. Third, we reasoned that this format could easily be paired with in-person individual or group therapy where such resources are available. In considering frequency of delivery, although once a week was most highly endorsed, a recent meta-analysis showed that psychotherapy delivered twice a week was more effective.^13^ Therefore, the 11 modules in the program are intended to be accessed over two menstrual cycles but concentrated in the least symptomatic phase of each cycle, which translated to approximately two modules per week. This program structure is based on patient advisor feedback that learning new skills would be difficult during the symptomatic premenstrual phase.

The strength of our current study is the inclusion of patient advisors which helped us develop a comprehensive list of symptoms—including suicidality—and education topics for the survey. Moreover, our survey captured interests from a global cohort of PMDD patients. However, our study has some limitations. While we acknowledge the recruitment of patients through IAPMD—a non-profit organization that has dedicated its efforts to educating the PMDD community for the past decade—it is possible that our sample was biased. Though the racial diversity accurately reflects the overall populations of the countries of our survey responders, our overall conclusions, drawn from a majority White sample, may not apply to racialized minority groups. Additionally, participants provided a self-reported diagnosis of PMDD which was not verified by prospective symptom tracking. We also acknowledge that participants may have had a higher awareness of PMDD, which may be associated with the reduced preference for basic topics like an overview of the menstrual cycle and hormone sensitivity, or PMDD diagnostic criteria, compared to the higher preference for less known topics such as the impact of life stages on PMDD symptoms.

## Conclusion

We report several patient-identified priority areas for a mental health program addressing PMDD. Overall, our findings support the need for combining techniques from DBT and CBT approaches. The inclusion of symptom tracking and psychoeducation alongside a PMDD-focused program can benefit patients in adopting positive practices that could cumulatively help in dealing with PMDD symptoms.^14^

## Supplemental Material

sj-docx-1-whe-10.1177_17455057251331697 – Supplemental material for Patient-identified priorities for a mental health program tailored to premenstrual dysphoric disorder Supplemental material, sj-docx-1-whe-10.1177_17455057251331697 for Patient-identified priorities for a mental health program tailored to premenstrual dysphoric disorder by Sneha Chenji, Christine Bueno, May Ly, Sandi MacDonald, Rebekka Chartier, Catherine Lunn and Jennifer L. Gordon in Women’s Health

sj-docx-2-whe-10.1177_17455057251331697 – Supplemental material for Patient-identified priorities for a mental health program tailored to premenstrual dysphoric disorder Supplemental material, sj-docx-2-whe-10.1177_17455057251331697 for Patient-identified priorities for a mental health program tailored to premenstrual dysphoric disorder by Sneha Chenji, Christine Bueno, May Ly, Sandi MacDonald, Rebekka Chartier, Catherine Lunn and Jennifer L. Gordon in Women’s Health

## Statements and Declarations

**Ethics approval and consent to participate:** The University of Regina Research Ethics Board was consulted prior to participant recruitment. Given that the purpose of this survey was solely to inform IAPMD’s efforts to improve its provision of mental health resources to its followers, this project was deemed to fall in the realm of quality assurance, therefore exempting it from ethical review (exemption provided on February 15th, 2024 per article 2.5 of the Tri-Council Policy Statement-2 of Canada). The survey was anonymous and did not collect any identifiable information from responders.

**Data availability:** The datasets generated during and/or analyzed during the current study are available from the corresponding author on reasonable request.

## References

[1] Reilly TJ Patel S Unachukwu IC , et al. The prevalence of premenstrual dysphoric disorder: Systematic review and meta-analysis. J Affect Disord 2024; 349: 534–540. 38199397 10.1016/j.jad.2024.01.066

[2] Hantsoo L Epperson CN. Premenstrual dysphoric disorder: epidemiology and treatment. Curr Psychiatry Rep 2015; 17(11): 87. 26377947 10.1007/s11920-015-0628-3 PMC4890701

[3] Gynecologists ACoOa. Management of premenstrual disorders: acog clinical practice guideline no. 7. Obstet Gynecol 2023; 142(6): 1516–1533. 37973069 10.1097/AOG.0000000000005426

[4] Hunter MS Ussher JM Cariss M , et al. Medical (fluoxetine) and psychological (cognitive-behavioural therapy) treatment for premenstrual dysphoric disorder: a study of treatment processes. J Psychosom Res. 2002; 53(3): 811–817. 12217456 10.1016/s0022-3999(02)00338-0

[5] Weise C Kaiser G Janda C , et al. Internet-based cognitive-behavioural intervention for women with premenstrual dysphoric disorder: A randomized controlled trial. Psychother Psychosom. 2019; 88(1): 16–29. 30783069 10.1159/000496237

[6] Nye A Delgadillo J Barkham M. Efficacy of personalized psychological interventions: A systematic review and meta-analysis. J Consult Clin Psychol. 2023; 91(7): 389–397. 37166831 10.1037/ccp0000820

[7] Eysenbach G. Improving the quality of web surveys: the checklist for reporting results of internet e-surveys (CHERRIES). J Med Internet Res 2004; 6(3): e34. 10.2196/jmir.6.3.e34 PMC1550605 15471760

[8] Matthews L Riddell J. Premenstrual dysphoric disorder (PMDD): the UK research agenda. University of the West of Scotland; 2023.

[9] Hernandez-Bustamante M Cjuno J , et al. Efficacy of dialectical behavior therapy in the treatment of borderline personality disorder: a systematic review of randomized controlled trials. Iran J Psychiatry 2024; 19(1): 119–129. 38420274 10.18502/ijps.v19i1.14347 PMC10896753

[10] Funnell EL Martin-Key NA Benacek J , et al. Preferences for and intention to use an app for premenstrual mental health symptoms using the Health Behaviour Model (HBM). npj Women’s Health 2024; 2(1): 18.

[11] Opatowski M Valdimarsdottir UA Oberg AS , et al. Mortality risk among women with premenstrual disorders in Sweden. JAMA Netw Open. 2024; 7(5): e2413394. 10.1001/jamanetworkopen.2024.13394 PMC11134214 38805225

[12] Osborn E Wittkowski A Brooks J , et al. Women’s experiences of receiving a diagnosis of premenstrual dysphoric disorder: a qualitative investigation. BMC Womens Health 2020; 20(1): 242. 33115437 10.1186/s12905-020-01100-8 PMC7594422

[13] Ciharova M Karyotaki E Miguel C , et al. Amount and frequency of psychotherapy as predictors of treatment outcome for adult depression: A meta-regression analysis. J Affect Disord 2024; 359: 92–99. 38777269 10.1016/j.jad.2024.05.070

[14] Song M Kanaoka H. Effectiveness of mobile application for menstrual management of working women in Japan: randomized controlled trial and medical economic evaluation. J Med Econ 2018; 21(11): 1131–1138. 30130990 10.1080/13696998.2018.1515082

