# Supplementary material for: Patient-identified priorities for a mental health program tailored to premenstrual dysphoric disorder
Source: Womens Health (Lond). 2025 Jun 13;21:17455057251331697. doi: 10.1177/17455057251331697 (PMC12166257; doi:10.1177/17455057251331697)
Supplement: Supplementary file 2 [file sj-docx-2-whe-10.1177_17455057251331697.docx]

PMDD project - Needs survey

Survey Flow

Standard: Survey information (5 Questions)

Block: Demographic Information (7 Questions)

Standard: Psychotherapy needs (6 Questions)

Standard: Content Delivery and Program Features (11 Questions)

| Page Break |  |
| --- | --- |

Start of Block: Survey information

Q1 The International Association for Premenstrual Disorders (IAPMD) aims to provide information and resources to anyone impacted by premenstrual disorders. One goal of ours is to increase access to effective mental health resources. We have crafted this survey in collaboration with researchers at the University of Regina to better understand what types of mental health resources may be helpful and what sort of content they should address.

The survey should take approximately **15-20 minutes to complete**. Your responses are completely anonymous and you can choose to quit the survey at any time or choose to skip any question you do not want to answer. Please note that while the primary purpose of this survey is to help us develop better mental health resources, the results of this survey may also be published in scientific manuscripts, presented at research conferences, and/or described on the IAPMD website. No identifying information is being collected; your identity will therefore remain completed anonymous.

Q2 Please click here before you proceed. 

| Page Break |  |
| --- | --- |

|  |
| --- |

Q3 Do you have premenstrual dysphoric disorder (PMDD) or premenstrual exacerbation (PME) of a mental health condition (self-diagnosed or otherwise)?

- Yes, I have PMDD  (1) 

- Yes, I have PME of an underlying condition; if so, which condition?  (2) __________________________________________________

- No, I don't have PMDD or PME  (3) 

Skip To: End of Survey If Q3 = No, I don't have PMDD or PME

|  |
| --- |

Q4 Which of the following are true regarding your diagnosis of PMDD or PME? Select all that apply.

- I have confirmed my diagnosis by tracking my daily mood for at least 2 cycles  (1) 

- I have confirmed my diagnosis by tracking my daily mood for 1 cycle  (2) 

- I have not tracked my symptoms but I see a pattern between my symptoms and my cycle  (3) 

- A health professional has officially diagnosed me with PMDD or PME  (4) 

- I've experienced symptom relief upon taking continuous birth control like Yaz or Yasmin or a GnRH analog treatment like Lupron or Zoladex  (5) 

| Page Break |  |
| --- | --- |

|  |
| --- |

Q5 Do you still actively experience symptoms of PMDD or PME?

- Yes, I still regularly experience symptoms  (1) 

- Yes, I still experience symptoms, but I experience some relief from a treatment. If so, please specify the treatment:  (2) __________________________________________________

- No, I have undergone chemical, natural, or surgical menopause  (3) 

- No, my PMDD or PME is effectively treated. If so, please specify the treatment:  (4) __________________________________________________

| Page Break |  |
| --- | --- |

End of Block: Survey information

Start of Block: Demographic Information

|  |
| --- |

Q6 In which country do you currently reside?

- Canada  (1) 

- US  (2) 

- Other, please specify:  (3) __________________________________________________

| Page Break |  |
| --- | --- |

|  |
| --- |

Q7 What is your age (in years)?

________________________________________________________________

|  |
| --- |

Q8 When did you first notice PMDD symptoms (age in years)?

________________________________________________________________

|  |
| --- |

Q9 How do you describe your gender identity?

▼ Woman (1) ... Prefer not to answer (6)

| Page Break |  |
| --- | --- |

|  |
| --- |

Q10 Please indicate which category(ies) best
describes your race or racial background. Please check all that apply to you:

- White / Caucasian (e.g., European descent)  (1) 

- Black (e.g., African, African Canadian, Afro-Carribean descent)  (2) 

- Latin American (e.g., Hispanic or Latin American descent)  (3) 

- East Asian (e.g., Chinese, Japanese, Korean, Taiwanese descent)  (4) 

- Indigenous (e.g., First nations, Inuk/Inuit, Métis)  (5) 

- Middle eastern (e.g., Arab, Persian, West Asian descent such as Afghan, Egyptian, Iranian, Kurdish, Lebanese, Turkish)  (6) 

- South Asian (e.g., Bangladeshi, Indian, Indo-Caribbean, Pakistani, Sri Lankan)  (7) 

- Southeast Asian (e.g., Cambodian, Filipino, Indonesian, Thai, Vietnamese)  (8) 

- Another race category, please specify:  (9) __________________________________________________

- Do not know  (10) 

- Prefer not to answer  (11) 

| Page Break |  |
| --- | --- |

|  |
| --- |

Q11 We want to make sure we survey people from a wide range of backgrounds. For that reason, we’d like to know the highest level of school you have completed or the highest degree you have received?

▼ Less than high school degree (1) ... Prefer not to answer (9)

|  |
| --- |

Q12 Which of these categories best describe your entire household income in the last year? (Include income, before taxes, from all sources, wages, rent from property, social security).

▼ Less than $10,000 (1) ... Prefer not to respond (14)

End of Block: Demographic Information

Start of Block: Psychotherapy needs

|  |
| --- |

Q13 At any given point in your life, have you received counselling?

- Yes, I have in the past  (1) 

- Yes, I am currently in counselling  (2) 

- No, never  (3) 

Display This Question:

If Q13 = Yes, I have in the past

Or Q13 = Yes, I am currently in counselling

|  |
| --- |

Q14 Do you know what type of counselling you've received or are currently receiving?

- Cognitive behavioral therapy (CBT)  (1) 

- Dialectical behavioral therapy (DBT)  (2) 

- Acceptance and commitment therapy (ACT)  (3) 

- Interpersonal psychotherapy (IPT)  (4) 

- Mindfulness-based therapy  (5) 

- Psychodynamic psychotherapy  (6) 

- Mixed approach (also called 'eclectic')  (7) 

- Counselling  (8) 

- Other, please specify  (9) __________________________________________________

- I don't know  (10) 

| Page Break |  |
| --- | --- |

| Page Break |  |
| --- | --- |

|  |
| --- |

Q15 Which of the following **symptoms** would you personally consider most important to address in a PMDD / PME focused program?

|  | Not at all important (1) | Slightly important (2) | Moderately important (3) | Very important (4) | Extremely important (5) |
| --- | --- | --- | --- | --- | --- |
| Extreme irritability (1) |  |  |  |  |  |
| Rage (2) |  |  |  |  |  |
| Extreme negative thoughts (3) |  |  |  |  |  |
| Thoughts of hurting oneself (4) |  |  |  |  |  |
| Thoughts of hurting others (19) |  |  |  |  |  |
| Thoughts of ending my life (18) |  |  |  |  |  |
| Hopelessness (5) |  |  |  |  |  |
| Inability to enjoy normally pleasurable activities (6) |  |  |  |  |  |
| Low self-esteem (7) |  |  |  |  |  |
| Anxious symptoms (8) |  |  |  |  |  |
| Lack of motivation (9) |  |  |  |  |  |
| Extreme lethargy or a lack of energy (20) |  |  |  |  |  |
| Feeling emotionally numb (10) |  |  |  |  |  |
| Conflicting thoughts and feelings (11) |  |  |  |  |  |
| Dealing with traumatic memories (12) |  |  |  |  |  |
| Dealing with dread and anxiety of upcoming symptoms (21) |  |  |  |  |  |
| Coping with guilt and shame (22) |  |  |  |  |  |
| Coping with brain fog (26) |  |  |  |  |  |
| Impulsive thoughts and actions (13) |  |  |  |  |  |
| Managing physical symptoms (e.g., bloating, breast tenderness, pain) (14) |  |  |  |  |  |
| Insomnia or night sweats (23) |  |  |  |  |  |
| Obsessive-compulsive thoughts (24) |  |  |  |  |  |
| Delusional thoughts (25) |  |  |  |  |  |
| Binge eating (15) |  |  |  |  |  |
| Alcohol and other drug use, please specify which substance: (16) |  |  |  |  |  |
| Other (17) |  |  |  |  |  |

| Page Break |  |
| --- | --- |

|  |
| --- |

Q16 Which of the following **social consequences** of PMDD / PME would you personally consider important to address in a PMDD / PME focused program?

|  | Not at all important (1) | Slightly important (2) | Moderately important (3) | Very important (4) | Extremely important (5) |
| --- | --- | --- | --- | --- | --- |
| Relationship with romantic partner (1) |  |  |  |  |  |
| Relationship with children (2) |  |  |  |  |  |
| Relationship with family (3) |  |  |  |  |  |
| Relationship with with friends (4) |  |  |  |  |  |
| Navigating social life (5) |  |  |  |  |  |
| Interacting with colleagues (6) |  |  |  |  |  |
| Interacting with healthcare providers (7) |  |  |  |  |  |
| Other (9) |  |  |  |  |  |

| Page Break |  |
| --- | --- |

|  |
| --- |

Q17 Which of the following would you personally prefer to use in a PMDD / PME focused program?

|  | Not at all (1) | Slightly prefer (2) | Moderately prefer (3) | Very much prefer (4) | Highly prefer (5) |
| --- | --- | --- | --- | --- | --- |
| Mindfulness-based techniques (1) |  |  |  |  |  |
| Lifestyle-related changes (2) |  |  |  |  |  |
| Links or books for additional reading (3) |  |  |  |  |  |
| Journaling (4) |  |  |  |  |  |
| Techniques to manage emotions (5) |  |  |  |  |  |
| Techniques to manage thoughts (6) |  |  |  |  |  |
| Techniques to manage actions / behavior (7) |  |  |  |  |  |
| Creating a self-care plan (8) |  |  |  |  |  |
| Creating a safety plan (9) |  |  |  |  |  |
| Creating a well-being to-do list (10) |  |  |  |  |  |
| Planning medical appointments (11) |  |  |  |  |  |
| Providing evidence-based information (15) |  |  |  |  |  |
| Information about how to connect with others with PMDD / PME (16) |  |  |  |  |  |
| Relaxation techniques (12) |  |  |  |  |  |
| Other, please specify (13) |  |  |  |  |  |

|  |
| --- |

Q18 Apart from content directly related to mental health, what other information about PMDD/PME do you think we should include in a PMDD / PME focused program?

|  | Not at all important (1) | Slightly important (2) | Moderately important (3) | Very important (4) | Extremely important (5) |
| --- | --- | --- | --- | --- | --- |
| Overview of hormones and the menstrual cycle (1) |  |  |  |  |  |
| Diagnostic criteria for PMDD & PME (2) |  |  |  |  |  |
| Molecular changes underlying symptoms (3) |  |  |  |  |  |
| Impacts of life stage on symptoms (e.g., pregnancy, menopause transition) (4) |  |  |  |  |  |
| Genetic contributions to PMDs (5) |  |  |  |  |  |
| Risk factors for PMDs (6) |  |  |  |  |  |
| Symptom tracking and scoring (7) |  |  |  |  |  |
| Treatment options with strong evidence (8) |  |  |  |  |  |
| Treatment options with promising evidence (9) |  |  |  |  |  |
| Treatment options with limited evidence (e.g., supplements) (10) |  |  |  |  |  |
| Lifestyle changes to help symptoms (11) |  |  |  |  |  |
| Rights in the workplace (13) |  |  |  |  |  |
| Other (12) |  |  |  |  |  |

End of Block: Psychotherapy needs

Start of Block: Content Delivery and Program Features

Q19 We would like to know more about your preferences for content format and features that would help in effectively engaging with mental health resources.

| Page Break |  |
| --- | --- |

|  |
| --- |

Q20 Which of the following **content formats** would you prefer for a PMDD / PME focused program?

|  | Not at all (1) | Slightly prefer (2) | Moderately prefer (3) | Very much prefer (4) | Highly prefer (5) |
| --- | --- | --- | --- | --- | --- |
| Written content (1) |  |  |  |  |  |
| Audio content (2) |  |  |  |  |  |
| Video content (3) |  |  |  |  |  |

| Page Break |  |
| --- | --- |

Display This Question:

If Q20 = Written content [ Slightly prefer ] 

Or Q20 = Written content [ Moderately prefer ] 

Or Q20 = Written content [ Very much prefer ] 

Or Q20 = Written content [ Highly prefer ] 

|  |
| --- |

Q21 Please drag and drop items below to rank your preferred **digital platform** for *written* content (1 = most preferred and 5 = least preferred):

______ Mobile app (1)

______ Email (2)

______ Website / blog style (3)

______ Hard copy/ printable workbook (4)

______ Other, please specify: (5)

| Page Break |  |
| --- | --- |

Display This Question:

If Q20 = Audio content [ Slightly prefer ] 

Or Q20 = Audio content [ Moderately prefer ] 

Or Q20 = Audio content [ Very much prefer ] 

Or Q20 = Audio content [ Highly prefer ] 

|  |
| --- |

Q22 Please drag and drop items below to rank your preferred **digital platform** for *audio* content (1 = most preferred and 5 = least preferred):

______ Podcast style (1)

______ Mobile app (2)

______ Website (3)

______ Audiobook (4)

______ Other, please specify: (5)

| Page Break |  |
| --- | --- |

Display This Question:

If Q20 = Video content [ Slightly prefer ] 

Or Q20 = Video content [ Moderately prefer ] 

Or Q20 = Video content [ Very much prefer ] 

Or Q20 = Video content [ Highly prefer ] 

|  |
| --- |

Q23 Please drag and drop items below to rank your preferred **digital platform** for *video* content (1 = most preferred and 5 = least preferred):

______ Mobile app (1)

______ Website (2)

______ Facebook (3)

______ Instagram (4)

______ Other, please specify: (5)

| Page Break |  |
| --- | --- |

|  |
| --- |

Q24 Which of the following **features** would you prefer to help you engage effectively in a PMDD / PME focused program?

|  | Not at all (1) | Slightly prefer (2) | Moderately prefer (3) | Very much prefer (4) | Highly prefer (5) |
| --- | --- | --- | --- | --- | --- |
| Video testimonials (2) |  |  |  |  |  |
| Written testimonials (3) |  |  |  |  |  |
| Self-help exercises (4) |  |  |  |  |  |
| Integrating symptom tracking in the program (5) |  |  |  |  |  |
| Humor-related content (e.g., memes) (6) |  |  |  |  |  |
| Individual peer support (7) |  |  |  |  |  |
| Group peer support (13) |  |  |  |  |  |
| Infographics (9) |  |  |  |  |  |
| Reminders (10) |  |  |  |  |  |
| Access to a professional (8) |  |  |  |  |  |
| Other, please specify: (11) |  |  |  |  |  |

| Page Break |  |
| --- | --- |

Display This Question:

If Q24 = Individual peer support [ Slightly prefer ] 

Or Q24 = Individual peer support [ Moderately prefer ] 

Or Q24 = Individual peer support [ Very much prefer ] 

Or Q24 = Individual peer support [ Highly prefer ] 

|  |
| --- |

Q25 Please indicate the type of peer support you would like:

|  | Not at all (1) | Slightly prefer (2) | Moderately prefer (3) | Very much prefer (4) | Highly prefer (5) |
| --- | --- | --- | --- | --- | --- |
| Complete the program with a peer (1) |  |  |  |  |  |
| Complete the program with a group of peers (2) |  |  |  |  |  |
| Self-paced, but include a chat feature to interact with peers in the program (3) |  |  |  |  |  |
| Self-paced, but include opportunities to socialize with peers (e.g., online coffee meeting) (4) |  |  |  |  |  |
| Other, please specify: (5) |  |  |  |  |  |

| Page Break |  |
| --- | --- |

|  |
| --- |

Q26 If we were to create an online structured mental health program for PMDD / PME...

How long would you like the entire program to be? Please drag and drop items below to rank your preferred **duration of the program** (1 = most preferred and 4 = least preferred):

______ Less than 8 weeks (1)

______ 8 to 12 weeks (2)

______ Self-paced (3)

______ Other, please specify (4)

| Page Break |  |
| --- | --- |

|  |
| --- |

Q27 How **frequently** would you want to be sent new program content? 

- Daily  (1) 

- Once a week  (2) 

- Once a month  (3) 

- Twice a month  (4) 

- Self-guided (e.g., second module becomes available upon completion of the first, with the option to save your progress)  (5) 

- Other, please specify  (6) __________________________________________________

| Page Break |  |
| --- | --- |

Display This Question:

If Q27 = Daily

And Q27 = Once a week

And Q27 = Once a month

And Q27 = Twice a month

And Q27 = Other, please specify

|  |
| --- |

Q28 How much average time would you be willing to devote to the program per week?

- 5-10 minutes or less  (1) 

- 15-20 minutes  (2) 

- 30-40 minutes  (3) 

- 40-60 minutes  (4) 

- A few hours  (5) 

- Other, please specify  (6) __________________________________________________

| Page Break |  |
| --- | --- |

Q29 Thank you so much for taking the time to answer our questions! 

Please make sure that you click the "Submit" button on the next page to complete your survey.

End of Block: Content Delivery and Program Features
